# 

**Published:** 1806-04

**Authors:** 


### LIST OF NEW MEDICAL PUBLICATIONS.

The Anatomy and Diseases of the Human Ear; by J. C. Saunders; with Plates by Heath; magnificent folio, 25s. in boards.

A Manual of Inoculation, for the Use of the Faculty and private Families; pointing out the most approved Method of Inoculating and conducting Patients through the Small-pox; extracted from the Writings of Dimsdale, Sutton, and other eminent Practitioners; by G. Lipscomb, Surgeon, 1s.

A Practical Treatise on various Diseases of the Abdominal Viscera; by C. R. Pemberton, M. D. F. R. S.; 8vo. 7s. boards.

Motherby's, or the New London Medical Dictionary, 4to. Part I. a new edition, to be compleated in Four Parts, 24s. boards.

An Essay on the Diseases incident to India Seamen and Lascars in long Voyages, by W. Hunter; folio, 15s.

A Treatise on Epilepsy, by H. Fraizer, M. D. 8vo. 2s. 6d.

A New System of Family Medicine, by W. Keighley, M. D. 8vo. 6s.

The Modern Practice of Physic, by E. G. Clarke, M. D. 8vo. 9s.

---

### TO CORRESPONDENTS.

Mr. Young's Letter to Dr. Adams, on the Hydatid Existence of Cancer, will appear in our next Number.

Communications are also received from Dr. James Clarke, Dr. Walker, and Mr. Robertson.

